# Structure and diffusive dynamics of aspartate α-decarboxylase (ADC) liganded with d-serine in aqueous solution†

**DOI:** 10.1039/d2cp02063g

**Published:** 2022-08-11

**Authors:** Tushar Raskar, Stephan Niebling, Juliette M. Devos, Briony A. Yorke, Michael Härtlein, Nils Huse, V. Trevor Forsyth, Tilo Seydel, Arwen R. Pearson

**Affiliations:** Institut Max von Laue – Paul Langevin 71 Avenue des Martyrs Grenoble 38000 France tforsyth@ill.eu seydel@ill.eu; Partnership for Structural Biology 71 Avenue des Martyrs Grenoble 38000 France; Institute for Nanostructure and Solid State Physics, Hamburg Centre for Ultrafast Imaging, Universität Hamburg Luruper Chaussee 149 Hamburg 22761 Germany arwen.pearson@cfel.de; European Molecular Biology Laboratory, Hamburg, Notkestr. 85 22607 Hamburg Germany; School of Chemistry and Bioscience, University of Bradford Bradford BD7 1DP UK; Faculty of Natural Sciences, Keele University Staffordshire ST5 5BG UK

## Abstract

Incoherent neutron spectroscopy, in combination with dynamic light scattering, was used to investigate the effect of ligand binding on the center-of-mass self-diffusion and internal diffusive dynamics of *Escherichia coli* aspartate α-decarboxylase (ADC). The X-ray crystal structure of ADC in complex with the d-serine inhibitor was also determined, and molecular dynamics simulations were used to further probe the structural rearrangements that occur as a result of ligand binding. These experiments reveal that d-serine forms hydrogen bonds with some of the active site residues, that higher order oligomers of the ADC tetramer exist on ns–ms time-scales, and also show that ligand binding both affects the ADC internal diffusive dynamics and appears to further increase the size of the higher order oligomers.

## Abstract 2

Neutron spectroscopy, dynamic light scattering, X-ray diffraction, and MD-simulations were used to investigate the effect of ligand binding on the structure and diffusive dynamics of *Escherichia coli* aspartate alpha-decarboxylase.

## Graphical abstract

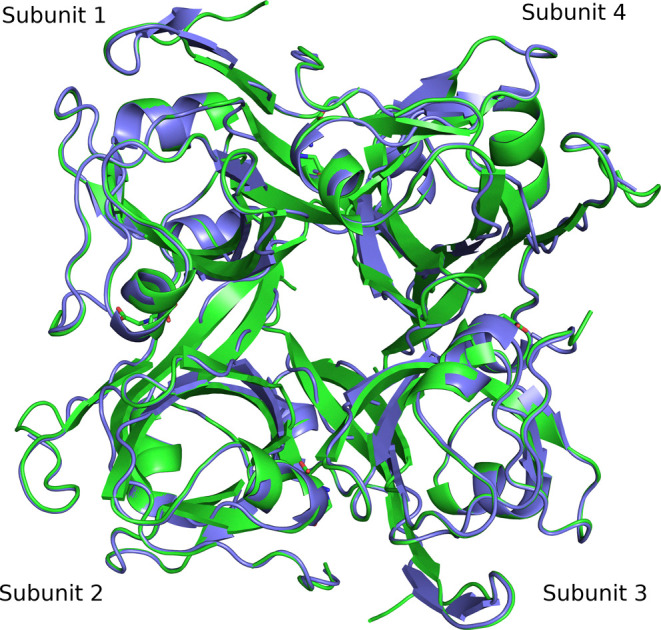

## 1 Introduction

It is becoming increasingly clear that an understanding of the structure–function relationships of biological macromolecules, such as enzymes, requires both a knowledge of their structure and of their dynamics. Enzymes, biological catalysts, are extremely large compared to chemical catalysts and are capable of very high specificity and selectivity, steering and controlling chemical reactions to a specific outcome. Although the active site, where catalysis occurs, is usually compact and localised, the whole enzyme contributes to the enzymatic reaction.^1,2^ Biological macromolecules can be considered as a soft elastic network and exhibit dynamics ranging over many orders in time, from femtosecond chemical reaction steps to much slower millisecond and second large scale conformational rearrangements.^3^ How these dynamics couple together to allow the slow motions of the protein to modulate catalysis remains an open question in structural enzymology and biophysics.^4,5^ There are multiple ways to probe dynamics, including experiments that examine the macromolecule as it proceeds through its reaction cycle,^6,7^ as well as methods that probe the equilibrium or non-driven dynamics.^8^ However, many of the experimental tools to probe equilibrium dynamics require the incorporation of labels, can access only dilute macromolecular suspensions, or introduce susceptibility to radiation damage.^9,10^ Quasi-elastic neutron scattering (QENS) can probe the functionally relevant intra- and inter-molecular dynamics of proteins,^11–13^ and their response to ligand binding^14,15^ or covalent modification.^16^ These studies have shown that the binding of a substrate or an analogue can lead not only to local structural rearrangements in the binding site, but also to a larger change in the overall intra- and the inter-molecular dynamics of the whole protein. Previously, QENS studies on aqueous solution samples have mainly employed abundant proteins available off-the-shelf, due to the large amounts of sample required.^11^

In the present work, we study the *Escherichia coli* enzyme aspartate α-decarboxylase (ADC), establishing a framework to test the effect of parameter changes in proteins on their dynamics. Here, this parameter is the presence or absence of the ligand d-serine. First, we determine the crystal structure of ADC in the presence of the ligand. Second, we link this structural information to results from molecular dynamics (MD) simulations. Third, we determine the pico- and nanosecond internal and center-of-mass diffusion of ADC in aqueous suspensions and discuss these results in relation to the structure. Our neutron spectroscopy experiments simultaneously probe spatial and time correlations, and the probed spatial scales are similar to those accessed by the X-ray diffraction experiment.

ADC catalyzes the oxidative decarboxylation of l-aspartate to yield β-alanine.^17^ β-Alanine is required for the biosynthesis of pantothenate (vitamin B5)^18^ which is then further converted to the important metabolic cofactor, Coenzyme A.^17^ ADC contains a covalently linked protein-derived pyruvoyl cofactor which forms a Schiff base with the substrate^19^ to initiate the decarboxylation reaction. This protein-derived cofactor is formed *via* the post-translational cleavage of the ADC zymogen protein backbone into α and β chains, resulting in the formation of a new C-terminus on the α chain and a N-terminal pyruvoyl cofactor on the β chain.^19,20^ The α C-terminus is extremely flexible, and the initial binding of the substrate is associated with its rearrangement to close over the active site. This conformational rearrangement is believed to play a role in determining the overall rate of catalysis.^21^d-serine is an inhibitor of ADC that, like the substrate l-aspartate binds in the enzyme active site.^22^

## 2 Experiments and methods

### 2.1 Sample preparation

ADC was expressed and purified according to previously published protocols.^17,19^ The final purified protein was concentrated using a 10 kDa molecular weight cutoff centrifugal unit until a concentration of 135 mg mL^−1^ was reached. The buffer used for the final concentration was 50 mM tris–HCl pH 7.4, 100 mM NaCl and 0.1 mM DTT in H_2_O. These solutions were dialysed against a fully deuterated buffer with the same composition for QENS measurements.

### 2.2 Protein concentration

Protein concentration was determined from absorbance at 280 nm using a DeNovix DS-11 spectrophotometer at the temperature *T* = (298.5 ± 0.5) K. The accuracy of the spectrophotometric measurements was confirmed by dialysis against 20 mM ammonium acetate, pH 7.0, 100 mM NaCl, lyophilyzing and weighing a known amount of ADC. Lyophilization was carried out using a Martin Christ instrument at a vacuum pressure of 0.06 mbar.

### 2.3 Crystallization of ADC and soaking with d-serine

The protein was concentrated to 10 mg mL^−1^ and was crystallized by vapour diffusion after mixing the protein and the precipitant in 1 : 1 ratio. The best crystals were obtained in 1.8 M ammonium sulphate, 100 mM sodium citrate pH 4.5. d-Serine was dissolved in the crystallization buffer to a concentration of 1 M before being added in a 1 : 1 molar ratio to the crystallisation droplet. The crystals were soaked for approximately 2 minutes before being washed in fresh crystallization buffer and then cryoprotected in crystallization buffer containing 20% v/v glycerol and flash cooled in liquid nitrogen.

### 2.4 X-ray data collection, processing and model refinement

X-ray diffraction data were collected at 100 K at Diamond Light Source on beamline I24 using a Pilatus3 6M detector. Data were integrated, processed and scaled using XDS.^23^ The structure was solved using the molecular replacement method as implemented in the software PHASER of the CCP4 suite.^24^ The structure 1AW8 from the PDB was used as the search model after removing the ligands and the water molecules. Crystallographic refinement was carried out using REFMAC5^25^ from the CCP4 suite.^24^ COOT^26^ was used for real-space modelling.

### 2.5 Theoretical diffusion coefficients

HYDROPRO^27^ was used to calculate the dilute-limit diffusion coefficients of apo (*i.e.*, devoid of its ligand) and d-serine-bound ADC, respectively, employing the apo-ADC structure 1AW8^20^ and d-serine complex structure determined in this work. The partial specific volume of ADC was calculated as 0.70 cm^3^ g^−1^. The solvent viscosity for D_2_O was set to 0.01830, 0.01175, 0.00830 poise for the temperatures *T* = 280, 295 and 310 K, respectively.^28^

### 2.6 Dynamic light scattering (DLS)

Dynamic light scattering experiments were conducted on an ALV-7004 instrument, covering the scattering angles from 30 to 150°, for both apo-ADC and d-serine liganded ADC solutions in D_2_O, at the sample bath temperature *T* = (298.8 ± 0.05) K. The molar concentration of d-serine was 20 times that of the ADC tetramer for all liganded samples to ensure complete saturation of all binding sites. The ADC concentrations covered a range from 1 to 5 mg mL^−1^ (16.67 to 83.3 μM).

### 2.7 Neutron spectroscopy

Experiments were performed on solutions of ADC in D_2_O buffer using both the IN16B and IN5 cold neutron spectrometers at the ILL.^29,30^ IN16B^31,32^ has an energy resolution of 0.75 μeV FWHM at 6.27 Å (Si(111) crystal analyzer configuration), and IN5 of approximately 80 μeV FWHM at 5 Å incident wavelength. Cylindrical double walled aluminium sample holders sealed with indium wire were employed, with the difference in the radius between the two walls being 0.15 mm and the outer diameter 22 mm. The total liquid sample volume was 1.2 mL. The identical samples were used consecutively in both the IN16B and associated IN5 experiments. The temperature was controlled with a standard Orange cryostat. For the neutron spectroscopy experiments, the total quantity of ADC used was 162.3 mg, corresponding to a dry protein volume fraction of 0.09.^33^ To ensure the complete saturation of all the ADC binding sites, 45 mg of d-serine was added per mL of the sample volume (135 mg mL^−1^ of ADC) corresponding to a total of 18.29 × 10^23^ protons from the d-serine *versus* 18.07 × 10^23^ protons from the ADC molecule, and to 47.6 molecules of d-serine per ADC monomer. The protein solution and pure D_2_O buffer reference samples were measured at *T* = 280, 295, and 310 K, respectively. For reference, pure d-serine at 45 mg mL^−1^ in D_2_O buffer solution was also measured on IN5 at *T* = 295 K. The Mantid software^34^ was used for the reduction of the IN16B data, and the Lamp software package^35^ provided by the ILL for the IN5 data. The empty container signal was subtracted from the IN16B spectra. All fits were carried out using *python3* scripts employing *scipy.optimize.curve_fit*. The fit parameter confidence bounds were calculated from the square root of the diagonal of the covariance matrix. The Voigt profiles used to calculate the scattering functions convoluted with the energy resolution functions were obtained from the real part of the Faddeeva function provided by *scipy.special*.

### 2.8 Molecular dynamics simulations

MD simulations were performed using Gromacs 2016.3^36–38^ with the Amber99SB-ILDN force field.^39^ Parameters for the d-serine ligand were derived from the existing l-Serine parameters. Charges for d-serine were obtained by the RESP approach, as described in ref. 40–42 Quantum mechanical calculations prior to RESP calculations were done with TURBOMOLE V7.1^43^ on the Hartree–Fock level using the RI-J approximation^44^ and a 6-31G* basis set.^45–47^ Two different side-chain conformers of d-serine were used and charges averaged over these two conformations.

For the pyruvate residue, existing force-field parameters from acetate and the amide carbonyl were used. Charges were calculated as described above using a single conformation. Force-field parameters for d-serine and the pyruvate residue are included as ESI.†

For analysis and visualisation of MD trajectories we used self-written *python* scripts in combination with the modules *MDAnalysis*,^48^*NumPy*^49^ and *Matplotlib*.^50^

The ADC-d-serine complex determined in this work and the apo-ADC structure (1AW8^20^) were used as the starting structures for the MD simulations. For each simulation, an ADC tetramer (apo and d-serine bound) was placed in a cubic box with periodic boundary conditions (1 nm initial minimum distance of protein to all boundaries). The box was filled with water (*ca.* 48 000 molecules). Some water molecules were replaced with sodium and chloride ions to reach a concentration of 100 mM of NaCl and to neutralize the negative charge of the protein. For each system, MD simulations were prepared at two different temperatures (285 K and 310 K) using the following protocol. After an energy minimisation (50 000 steps or maximum force <10 kJ mol^−1^ nm^−1^), an *NVT* equilibration with modified Berendsen thermostat, velocity rescaling^51^ and a 0.1 ps timestep (separate heat bath for protein and solvent + ions) was run. This was followed by a *NPT* equilibration using a Parrinello–Rahman pressure coupling^52,53^ at 1 bar with a compressibility of 4.5 × 10^−5^ bar^−1^ and a 2 ps time constant. During both equilibrations, a position restraint potential with a force constant of 1000 kJ mol^−1^ nm^−2^ was added to all protein atoms (including the ligand). All bonds to hydrogen atoms were constrained with the Linear Constrained Solver (LINCS)^54^ with an order of 4 and one iteration. Production MD simulations were run with a time step of 1 fs and the leap-frog integrator. Coordinates were saved every 10 ps. A grid-based neighbor list with a threshold of 1 nm was used and updated every 10 fs. For long-range electrostatic interactions above 1 nm the particle-mesh Ewald method^55,56^ was used with a fourth order interpolation and a maximum spacing for the FFT grid of 1.6 Å. Lennard-Jones interactions were cut-off above 1 nm. A long range dispersion correction for energy and pressure was used to compensate for the Lennard-Jones interaction cut-off.^37^ A total time of 250 ns was acquired for each of the four MD simulations.

## 3 Results

### 3.1 Crystal structure

The crystal structure of ADC in complex with d-serine was determined to a resolution of 1.9 Å and deposited in the protein data bank with the ID 7A8Y (Table 1). The structure was refined to final crystallographic *R*_work_ and *R*_free_ values of 17.1% and 18.7%, respectively (Table 1). As in the apo-ADC structure,^20^ the liganded ADC tetramer is formed by a crystallographic two-fold, with two ADC monomers in the asymmetric units. As in the apo structure, a fraction of mis-processed^57^ ADC is present, where the backbone of the zymogen is cleaved, but the β subunit has an N-terminal serine instead of a pyruvate. This mis-processed form is present at ≈40% occupancy.

**Table 1:** Crystallographic refinement statistics

	ADC-d-serine complex (PDBID 7A8Y)
Wavelength (Å)	0.9778
Resolution range (Å)	46.93–1.75 (1.81–1.75)
Space group	*P*6122
Unit cell (Å)	71.3, 71.3, 216.6, 90, 90, 120
Total number of reflections	67 840 (6601)
Number of unique reflections	33 921 (3301)
Multiplicity	2.0 (2.0)
Completeness (%)	99.99 (99.97)
Mean *I*/sigma(*I*)	21.50 (5.25)
Wilson *B*-factor (Å^2^)	19.07
*R*-merge	0.02384 (0.1564)
*R*-meas	0.03371 (0.2212)
*R*-p.i.m.	0.02384 (0.1564)
CC1/2	0.99 (0.93)
CC*	1.00 (0.98)
Reflections used in refinement	33 921 (3301)
Reflections used for *R*-free	1702 (162)
*R*-work	0.1712 (0.2110)
*R*-free	0.1871 (0.2266)
CC(work)	0.956 (0.836)
CC(free)	0.957 (0.807)
Number of non-hydrogen atoms	2173
Macromolecules	1951
Ligands	14
Solvent	208
Protein residues	244
RMS bonds (Å)	0.014
RMS angles (°)	1.64
Ramachandran favored (%)	96.51
Ramachandran allowed (%)	3.06
Ramachandran outliers (%)	0.44
Rotamer outliers (%)	3.0
Clashscore	4.38
Average *B*-factor (Å^2^)	23.04
Macromolecules	21.69
Ligands	27.72
Solvent	35.37

**Fig. 1:**
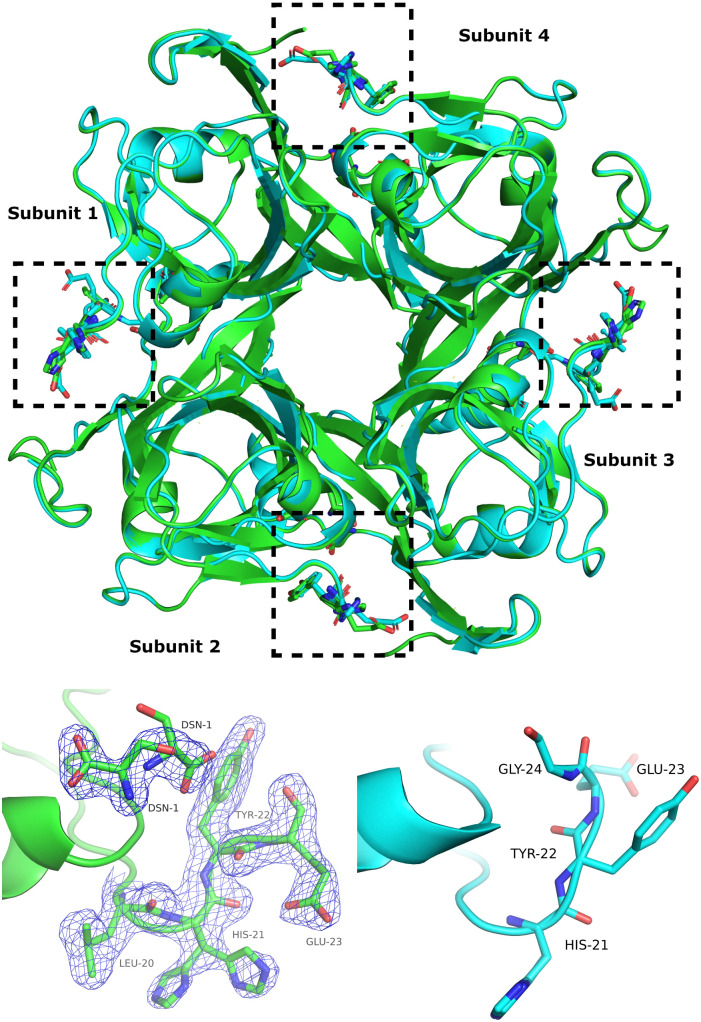
Top: Superposition of apo-ADC (PDBID 1AW8) (blue) and of the ADC-d-serine complex structure determined in this work (green). The active sites are indicated by dashed rectangular boxes. Bottom: Change in the conformations of the α C-terminal loop upon binding of d-serine in subunit 3 (bottom left, *cf.* dashed boxes in the top figure) and the corresponding loop conformation in apo-ADC (1AW8) (bottom right). The 2*mF*_o_−*DF*_c_ electron-density map is displayed at a contour level of 1*σ* where *m* denotes “figure of merit” and *D* “Sigma-A weighting factor”.

Comparison of the apo-ADC structure (1AW8) with the ADC-d-serine complex shows that the α C-terminal loop opens upon d-serine binding (Fig. 1).

The d-serine molecule adopts two conformations in both subunits of the asymmetric unit. One conformer (60% occupancy) forms hydrogen bonds with the main chain of ALA-75, the side-chains of ARG-54 and THR-57, and the pyruvoyl carbonyl (Fig. S1, ESI†) adopting a similar binding conformation to the native substrate l-aspartate. The second conformer (40% occupancy) forms hydrogen bonds with the side-chains of LYS-9 and TYR-58 (Fig. S1, ESI†) and with the nitrogen atom of the mis-processed β N-terminal SER-25.

In the apo-ADC structure (1AW8), residues 22–24 of the α C-terminal loop adopt two conformations, whereas in the d-serine complex, these residues adopt a single, open conformation (Fig. 1, bottom). The degree of “openness” is not the same in the two ADC subunits of the asymmetric unit. There is a significant change in the conformation of the α C-terminal loop of chain A (subunit 1) which shows a displacement of 4.3 Å of the C_α_ of GLU-23 from its position in the apo structure and a clear change in the conformation of its side-chain (Fig. 1, bottom). However, for chain D (subunit 2), the structural change is relatively small with a C_α_–C_α_ distance of only 0.8 Å for the same residue, although d-serine is bound in both subunits (Fig. 1, top).

### 3.2 MD simulations

**Fig. 2:**
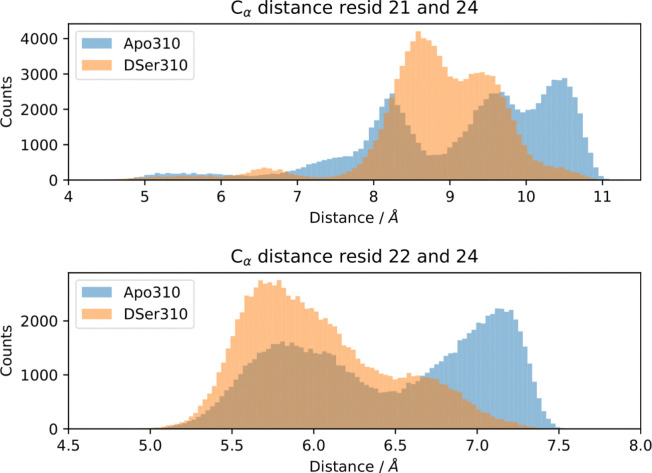
Histograms for the average C_α_–C_α_ distances for all the four subunits between HIS-21-GLY-24 (top) and between TYR-22-GLY-24 (bottom) for apo-ADC (blue) and d-serine liganded ADC (orange), simulated for *T* = 310 K.

The above observation from X-ray diffraction is supported by the MD simulations of apo and d-serine complexed ADC. The conformational change which is associated with the displacement of the C-terminal loop of subunit 3 occurs mainly between HIS-21 and GLY-24. Hence, we monitored the change in the C_α_–C_α_ distance between HIS-21 and GLY-24, and between TYR-22 and GLY-24. The distance histogram profiles (Fig. 2) are significantly different for apo-ADC and d-serine liganded ADC. The histogram profile on the top panel shows three peaks for the distance between HIS-21 and GLY-24 in apo-ADC at 8.2, 9.7 and 10.5 Å whereas for the d-serine complex, there are just two peaks at 8.7 and 9.5 Å. A plot of the distance between the HIS-21 and GLY-24 residues corroborates a change of the distance between these residues as a result of the ligand binding (Fig. S5 in the ESI†). Similarly, there is a clear shift in the distribution of distance between TYR-22 and GLY-24 from 7.25 Å in apo-ADC to 5.75 Å in the d-serine complex (bottom panel). These changes indicate that the motion of the C-terminal loop is more confined in the presence of the ligand than in apo-ADC. Further, the changes in the two distances for the individual subunits support the observation from the crystal structure that the influence of binding of d-serine on the dynamics of the C-terminal loop is neither completely symmetrical nor consistent among the four subunits (Fig. S2 in the ESI†).

### 3.3 Picosecond diffusive motions in solution

**Fig. 3:**
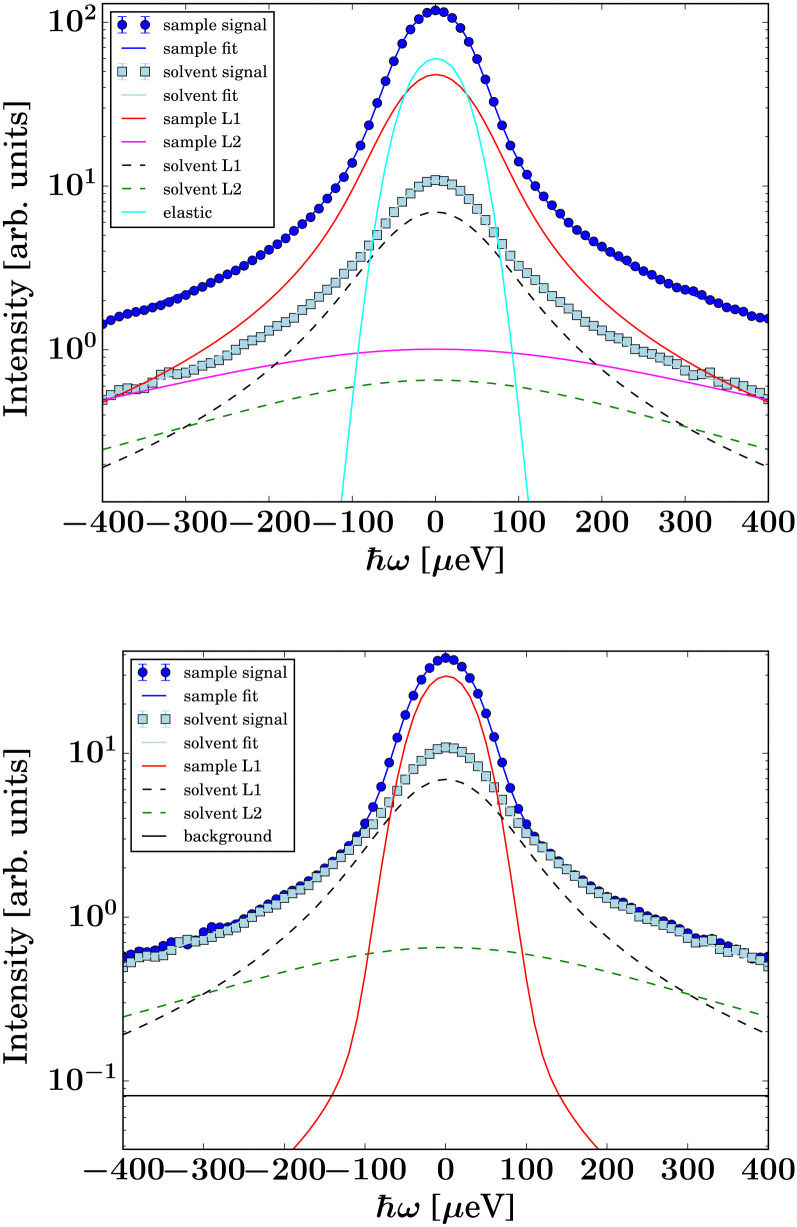
Example spectra (symbols) recorded on IN5 on d-serine liganded ADC (top, dark blue circles) and apo-ADC (bottom, dark blue circles), respectively, at *q* = 0.6 Å^−1^ at *T* = 295 K. The light blue square symbols denote the corresponding solvent signal. The lines superimposed on the protein sample spectra represent fits of eqn (2). The dashed lines represent the two Lorentzians describing the solvent, eqn (1). The narrow solid light blue line only visible in the top panel accounts for an apparent elastic contribution (
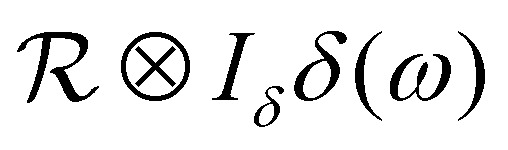
 in eqn (2)) that is only significantly present in the case of the liganded sample. This signal is synonymous with the spectrometer resolution. The red and magenta Lorentzians account for internal diffusive dynamics of the proteins. The broad magenta Lorentzian shows a significant presence only for liganded ADC. The straight black line only visible in the lower panel accounts for an apparent background.

The data from the neutron time-of-flight spectrometer IN5 contain information on both quasielastic scattering arising from diffusive motions and inelastic scattering arising from vibrational motions. Here, we present the QENS part only. The reduced QENS data from IN5 were fitted for each momentum transfer *q* independently in two steps. First, the spectra from the buffered D_2_O solvent were fitted by^58^1
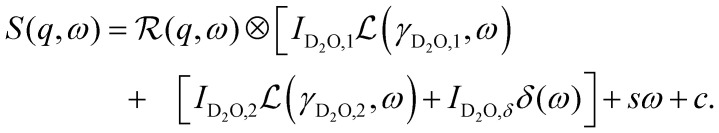
Therein, 
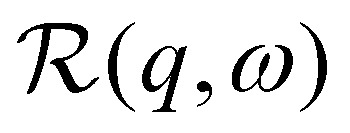
 represents the apparent energy resolution function of IN5, which also includes effects from the sample container geometry, and ⊗ the convolution in the energy transfer *ħω*. This convolution is carried out by modeling 
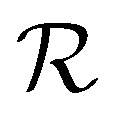
 as a sum of Gaussian functions, such that the observable *S*(*q*,*ω*) can be fitted by a sum of Voigt functions.^12,59,60^ (*σ*, ·) represents a Lorentzian function with the width *σ*, and *δ*(*ω*) the Dirac function describing the elastic scattering arising from the sample container. *I*_D_2_O,1,2,*δ*_, *s* and *c* denote *q*-dependent scalars, where *s* and *c* account for an apparent background arising from the sample, container, and instrument itself. The resulting components of the fits of the pure solvent signals according to eqn (1) are represented as dashed lines in Fig. 3.

Second, the QENS spectra from the protein solution samples and pure d-serine reference sample are fitted by2
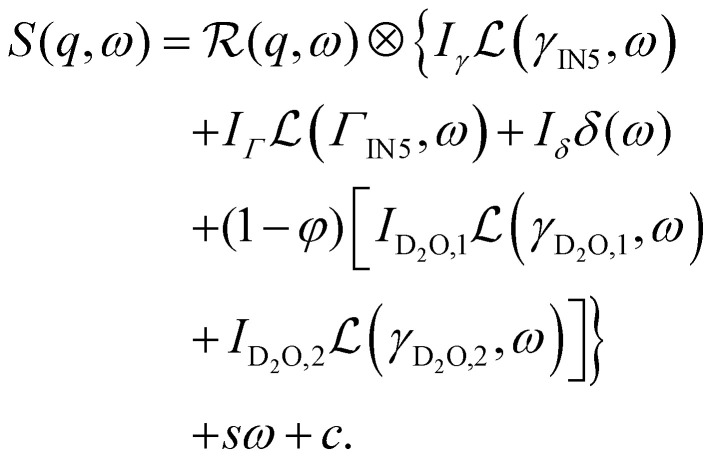
*I*_D_2_O,1,2_, *s*, and *c* are fixed from the fit results of the corresponding pure solvent (eqn (1)), and *φ* = 0.09 is the known protein volume fraction. The term *I*_*δ*_*δ*(*ω*) accounts for the apparent elastic scattering arising from both the sample container and sample dynamics that are quasi-static on the observation scale of IN5. The results of the fit of eqn (2) are depicted in Fig. 3 for one example spectrum each of liganded ADC and apo-ADC, respectively, along with both the protein and pure solvent spectra themselves. We note that not all lines indicating the individual components in eqn (2) are visible in both panels of Fig. 3 due to weak intensities below the *y*-axis limit.

**Fig. 4:**
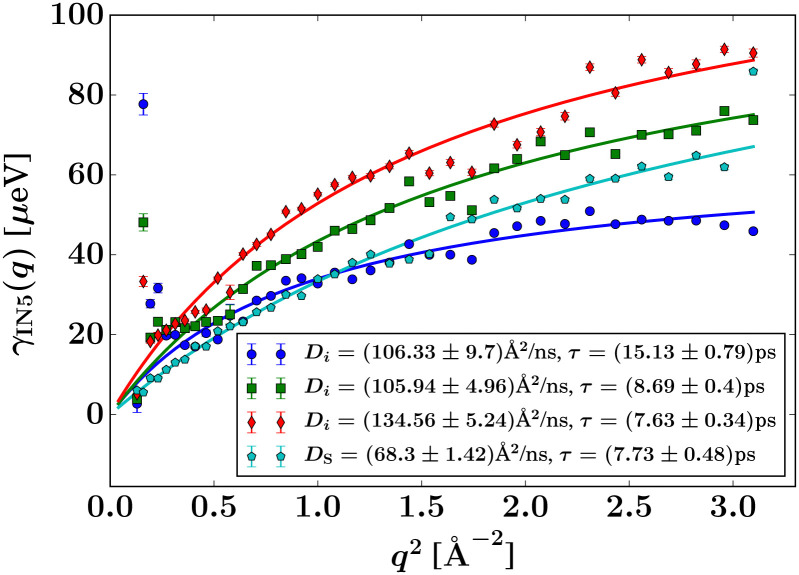
Width *γ*_IN5_ of the Lorentzian accounting for slow internal diffusive motions observed on IN5 (eqn (2)) for d-serine liganded ADC at different temperatures (circles: 280 K, squares: 295 K, and diamonds: 310 K) as well as for the pure d-serine reference sample (pentagrams: 295 K), and fits using the jump diffusion model (eqn (5)).

The signal of the apo-ADC sample is weaker compared to the signal from the liganded sample, which can be attributed to the addition of d-serine. In the case of the apo-ADC sample, the Lorentzian associated with the slower part of the internal diffusive dynamics becomes narrow and takes the role of the apparent elastic contribution (Fig. 3, bottom, red solid line). In contrast, this contribution is broad in the liganded sample (Fig. 3, top, red solid line). For this liganded sample, *γ*_IN5_(*q*) (eqn (2)) for all measured temperatures are summarized in Fig. 4. A pure d-serine solution reference sample was also measured at *T* = 295 K. For this sample, the linewidth is similar compared to the corresponding linewidth in the liganded ADC sample, but its *q*-dependence is qualitatively different. It should be noted that *γ*_IN5_ in eqn (2) accounts for an average over multiple dynamic contributions that cannot be further discerned with the current accuracy of the data and modeling. In the case of the ADC-d-serine sample, this Lorentzian likely reflects both bound and unbound d-serine. The observed diffusion coefficient of pure d-serine in solution is in reasonable agreement with earlier findings.^61–63^ It appears that few accessible diffusive dynamic contributions on the picosecond time scale are associated with the protein itself (Fig. 3, bottom), suggesting an overall highly rigid protein consistent with its high content of β-sheet (approximately 40%) as determined using the DSSP server.^64,65^ Further fit parameters from eqn (2) are included as ESI.†

### 3.4 Nanosecond internal diffusive motion

With its high energy resolution, the spectrometer IN16B accesses quasi-elastic scattering containing information on superimposed center-of-mass and internal diffusion. The scattering function observable on IN16B was modeled by:^12,59,60^3
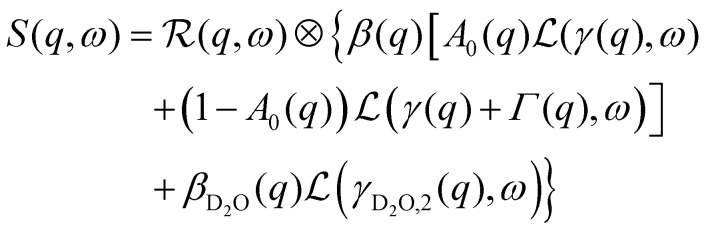


Therein, 
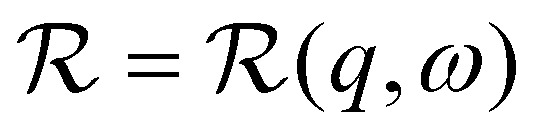
 denotes the spectrometer resolution function, and 
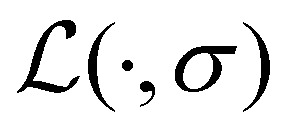
 is a Lorentzian function with the HWHM *σ*. *β*(*q*), *A*_0_(*q*), *γ*(*q*), and *Γ*(*q*) are scalar fit parameters. The scalar parameters for the solvent water contribution *β*_D_2_O_(*q*) and *γ*_D_2_O_(*q*) were fixed based on a pure solvent measurement using established protocols.^66^

By a global fit of the spectra for all *q* simultaneously using eqn (3), a Fickian center-of-mass diffusion of the proteins with the observable apparent diffusion coefficient *D* was assumed, as established for other proteins,^59,60,66^

4 *γ*(*q*) = *Dq*^2^.

**Fig. 5:**
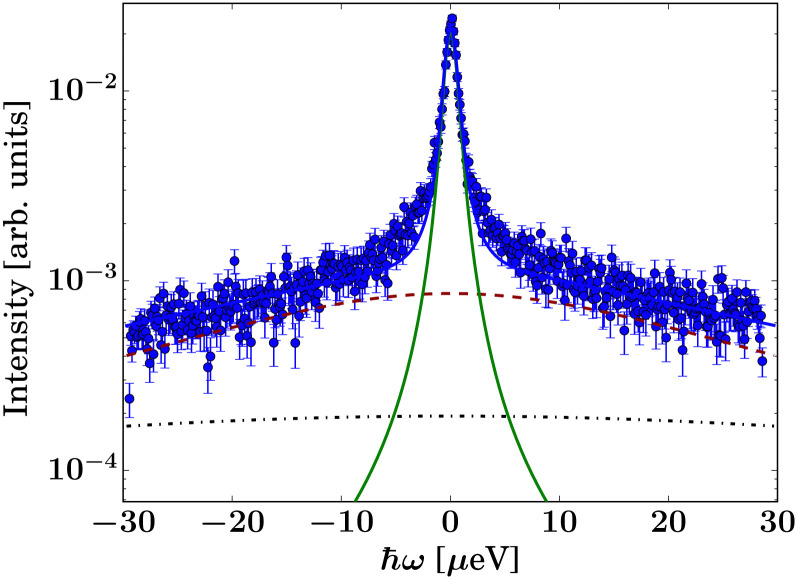
Example spectrum (symbols) of ADC-d-serine recorded on IN16B at *T* = 295 K, *q* = 0.78 Å^−1^ and fit using eqn (3) (solid line superimposed on the symbols). The fit consists of the Lorentzian contributions given by eqn (3), which are represented by the additional lines: the lower dot-dash black line denotes the solvent contribution, the solid dark green line the center-of-mass diffusion and the light green dashed line the internal diffusion of the proteins.

Therein, *D* = *D*(*D*_r_, *D*_t_) consists of contributions from both rotational *D*_r_ and translational *D*_t_ diffusion.^59,67^ Simultaneously, the internal diffusion was also obtained from eqn (3), assuming jump diffusion^68^ as reasonable approximation,^11^5
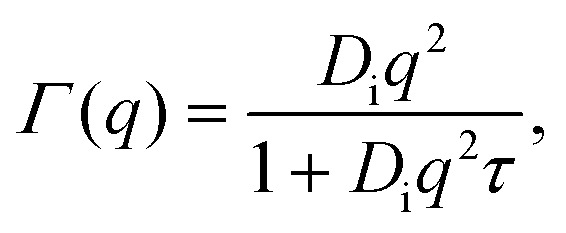
where *D*_i_ is the jump diffusion coefficient and *τ* is the so-called residence time between diffusive jumps. Importantly, in the fits of the IN16B spectra, the values of *D*_i_ and *τ* are fixed based on the results from IN5 for d-serine liganded ADC (Fig. 4). An example spectrum and fit using eqn (3) is shown in Fig. 5.

**Fig. 6:**
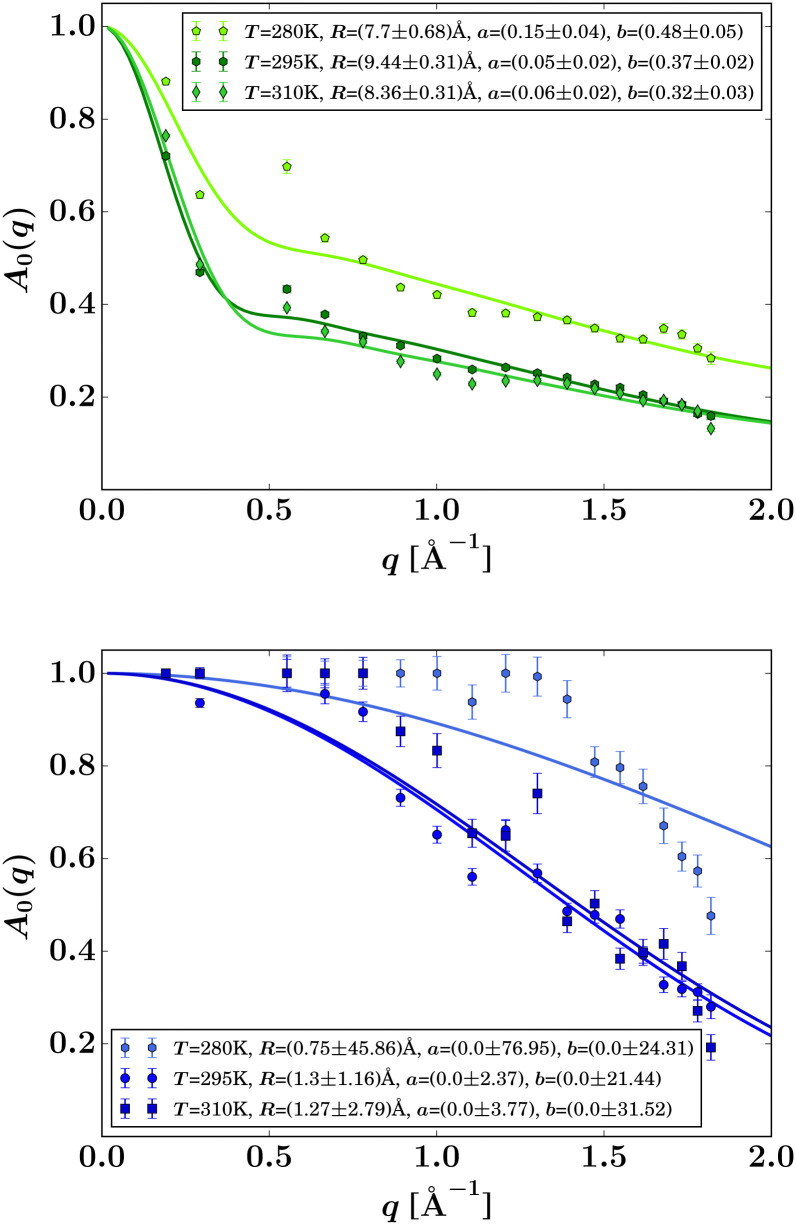
EISF *A*_0_(*q*) (symbols) for liganded ADC (top) and apo-ADC (bottom) obtained from eqn (3) when fixing *Γ* by using the IN5 result, and fits by eqn (6) (solid lines). The resulting fit parameters are given in the legends.

In eqn (3), *A*_0_(*q*) can be identified with the elastic incoherent structure factor EISF^69^ (Fig. 6) as follows:

6 *A*_0_(*q*) = *a* + (1 − *a*)(*bA*_3-jump_(*q*,*d*) + (1 − *b*)*A*_sphere_(*q*)),

where *a* is the fraction of scatterers within the protein that are immobile (apart from the protein center-of-mass diffusion) on the observation or coherence time of the measurement (≈4 ns for IN16B), and *A*_3-jump_(*q*,*d*) accounts for reorientational jumps between three equivalent sites associated with the methyl groups,7
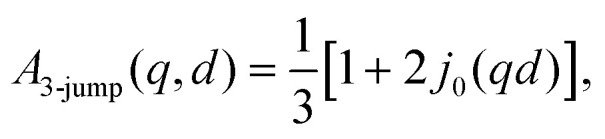
with the fixed jump length *d* = 1.715 Å for these methyl groups.^60,69,70^ It is further assumed that the protein side-chains diffuse on the surface of a sphere with the average radius *R*,^69^8
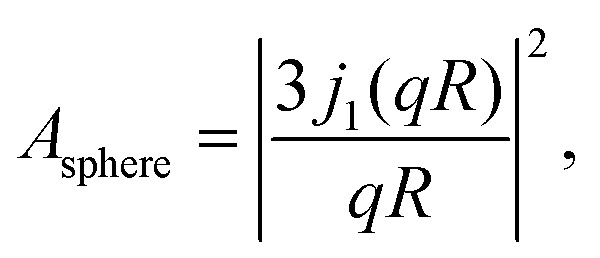
where *j*_0_ and *j*_1_ are the spherical Bessel functions of zeroth and first order, respectively. *b* denotes the relative weight of the contributions from *A*_3-jump_(*q*) and *A*_sphere_. Results for the EISF *A*_0_(*q*) and fits using eqn (6) are reported in Fig. 6 for both samples at *φ* = 0.09. Stable fits for the EISF for both samples can only be achieved by fixing the linewidth *Γ* in eqn (3) using the fit results from IN5. In contrast, when not fixing the internal dynamics (not shown), a finite internal linewidth *Γ*(*q*) in eqn (3) can only be seen for the samples with the ligands. For the apo-samples, *Γ*(*q*) → ∞ in the fits of the IN16B spectra. At the same time, the errors on the internal diffusion fit parameters diverge. This could suggest very fast internal motions beyond the dynamic window accessible by IN16B in the case of the apo-sample.

The internal diffusive dynamics seem to change substantially depending on whether or not the ligand is present, as reported earlier on a different system,^15^ although in the case of the ADC/d-serine system it cannot yet be ruled out that this change simply reflects the dynamics of the bound d-serine itself. Independently from the assumptions on the internal dynamics, the fit results for the center-of-mass diffusion coefficient *D* appear rather robust. The systematic error due to assumptions on the internal dynamics is on the order of ±0.5 Å^2^ ns^−1^, thus, larger than the fit parameter confidence bounds, but significantly smaller than the difference in the diffusion between the two samples (Table S2 in the ESI†).

### 3.5 Center-of-mass diffusion: QENS and DLS

The global apparent center-of-mass diffusion *D* obtained from fitting eqn (3) can be approximated by the Stokes–Einstein relation9
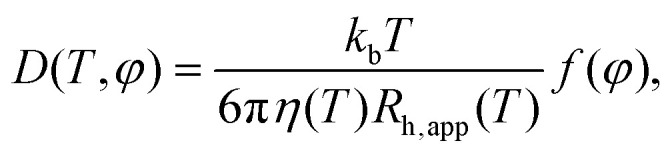
which would hold accurately for the translational diffusion, where *η* is the solvent viscosity and *R*_h,app_ the effective protein hydrodynamic radius. *f*(*φ*) is a scalar function of the protein volume fraction *φ* and does not depend on *T* or *R*_h,app_.^33,59,71^ Thus, *D* directly reflects the average hydrodynamic size of the diffusing particle, which can be a protein monomer, oligomer, or cluster.

**Fig. 7:**
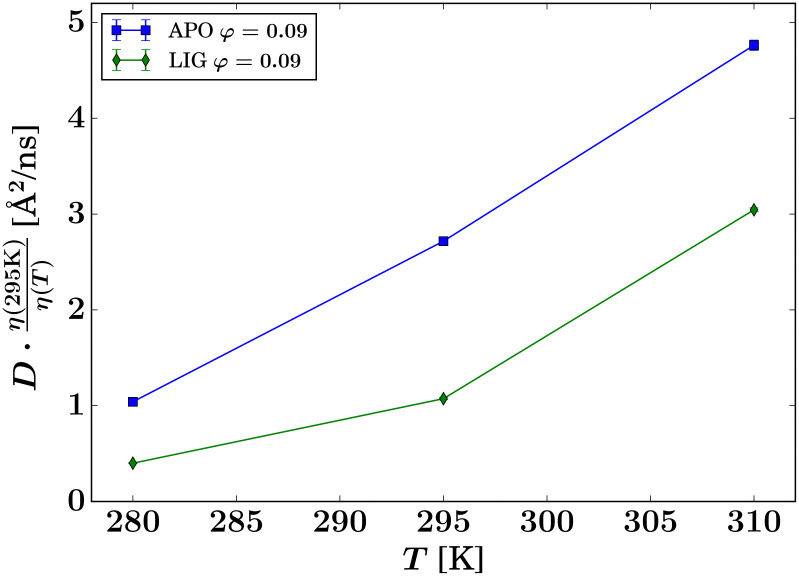
Observable apparent center-of-mass diffusion coefficients *D* (symbols) obtained from the IN16B spectra (protein volume fraction *φ* = 0.09, rescaled by the temperature-dependent solvent viscosity *η*(*T*)), *versus* sample temperature *T*. The lines are guides for the eye and do not represent any fit.

Interestingly, the results in Fig. 7 indicate that the hydrodynamic size of the ADC tetramer or aggregate depends strongly on whether or not ligand is present. For apo-ADC, the Stokes–Einstein dependence, eqn (9), can be observed, as illustrated by the linear dependence on *T*, suggesting that the size of the apo-ADC assembly is constant within the observed temperature range. In contrast, for d-serine liganded ADC, a larger assembly seems to be present, which could partially dissociate at higher temperatures, suggested by the slope-change in the rescaled *D* (Fig. 7). Since the crystal structure indicates that ligand binding does not significantly alter the hydrodynamic size of the ADC tetramer (Table S1 and Fig. S12 in the ESI†), the difference between apo-ADC and liganded ADC samples is best explained by the formation of a higher order protein oliogmer or cluster in solution in the presence of d-serine.^33,72^ This change in the hydrodynamic size by cluster formation is further corroborated by a plot of the apparent hydrodynamic radius calculated using eqn (9) (Fig. S13 in the ESI†) which, however, largely underestimates the actual hydrodynamic radius in the case of QENS which measures a function *D* = *D*(*D*_t_,*D*_r_) of the translational *D*_t_ and rotational *D*_r_ center-of-mass diffusion. The consequences thereof will be addressed later in this section.

This higher order oligomer or cluster formation in the presence of the ligand was further investigated by dynamic light scattering (DLS) measurements. DLS accesses the collective diffusion of relatively dilute samples as opposed to the short-time self-diffusion in concentrated samples accessed by spatially incoherent QENS. DLS also observes substantially longer diffusive time scales on the order of milliseconds as opposed to the nanosecond diffusive short-time regime explored by QENS. Due to the low momentum transfers, DLS in general only accesses the translational part *D*_t_ of the diffusion coefficient in the case of proteins. Examples of DLS autocorrelation functions and *q*-dependent decay rates are given in the ESI† (Fig. S10 and S11).

**Fig. 8:**
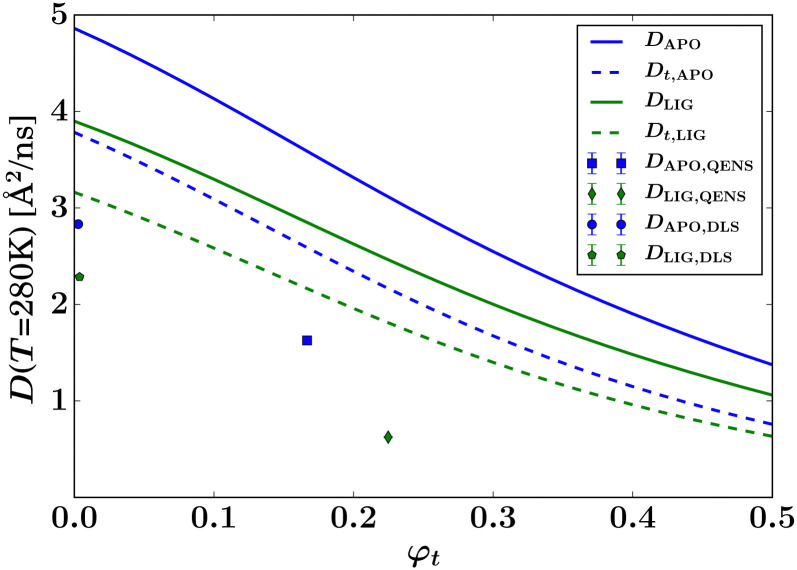
Summary of the QENS and DLS results for the global center-of-mass diffusion coefficient *D* of the proteins *versus* the theoretical volume fraction *φ*_t_ of effective hard spheres, at *T* = 280 K. For DLS, the symbols (at *φ* ≈ 0) represent the translational diffusion *D*_t_ probed by this method, rescaled to *T* = 280 K according to the Stokes–Einstein relation. For QENS, the symbols represent the apparent short-time self-diffusion consisting of contributions from both rotations and translations. The solid lines report the theoretical apparent diffusion of effective spheres with the hydrodynamic size of apo-ADC and d-serine liganded ADC tetramers, respectively. The dashed lines represent the corresponding translational diffusion (*cf.* legend).

By fitting the diffusion coefficients for all five measured concentrations for both the samples apo-ADC and d-serine liganded ADC, average diffusion coefficients were obtained from DLS in the low-concentration limit (Fig. 8, symbols at *φ*_t_ ≈ 0), amounting to (4.05 ± 0.02) and (4.30 ± 0.03) Å^2^ ns^−1^ for liganded ADC and apo-ADC, respectively. However, this difference in the diffusion coefficient reflects only a minor change in the hydrodynamic radius *R*_h_ from (4.28 ± 0.03) nm for ADC-APO to (4.20 ± 0.02) nm for the liganded form due to the different solvent viscosities. Being both larger than the calculated values of *R*_h_ for tetramers from HYDROPRO^27^ (2.96 and 3.28 nm, respectively, *cf.* Table S1 in the ESI†), these DLS values for *R*_h_ indicate the formation of small clusters in the nearly dilute limit (symbols at *φ*_t_ ≫ 0 in Fig. 8). For comparison with the experimental data, the theoretical diffusion coefficients at infinite dilution for the apo-ADC and d-serined-serine complex were also calculated using HYDROPRO^27^ (Table S1 in the ESI†). In general, viscosity depends on both the ionic concentration as well as on the temperature.^73–75^ Changes in viscosity upon addition of l-Serine have been studied previously^73–76^ and the change in viscosity is approximately 5–8% when the serine concentration is increased from 0 to 3.6 M.^75^ To account for this increase in viscosity in the calculation of the theoretical diffusion coefficient by HYDROPRO, we assumed a viscosity increased by 8% relative to the viscosity of pure D_2_O. The thus calculated translational diffusion coefficients for apo-ADC and liganded ADC are 3.784 and 3.416 Å^2^ ns^−1^, respectively, at 280 K, and 6.206 and 5.623 Å^2^ ns^−1^, respectively, at 295 K (*cf.* Table S1 in the ESI†). The theoretical dilute-limit translational *D*_t_ (corresponding to DLS measurements) and apparent *D* (corresponding to QENS measurements) diffusion coefficients were extrapolated to higher protein volume fractions using models for colloidal hard spheres^71^ (lines in Fig. 8), assuming the theoretical effective hydrodynamic volume fraction *φ*_t_ = *φ*(*R*_h_/*R*)^3^ calculated from the hydrodynamic *R*_h_ and dry *R* effective protein radii, which differ depending on whether or not the ligand is present.

Importantly, in Fig. 8, the DLS data compare with the dashed lines, and the QENS data with the solid lines in terms of the theory for diffusing tetramers. Notably, the lower experimental values from both DLS and QENS compared to the respective theoretical expectations for ADC tetramers indicate that the hydrodynamic size of the experimentally observed diffusing objects is larger than the crystallographic tetramer for both apo-ADC and the d-serine complex. This observation suggests that ADC forms higher order oligomers or clusters in solution both with and without d-serine, although in the presence of the ligand these oligomers or clusters are larger. This deduction can be carried even further when assuming compact spherical clusters, which renders a separation of the rotational and translational diffusion contributions in the cluster QENS signal possible^72^ (Fig. S14, ESI†). In this simplistic picture, the clusters would be large in the crowded situation measured by the QENS experiment, with 10 tetramer members in the case of the apo form, and approximately 58 tetramer members for the liganded form of ADC (Fig. S14, ESI†).

## 4 Discussion

Previous work on diffusive dynamics in protein solutions has mainly focused on abundant commercially available standard samples. Here, for the first time, the effect of ligand binding on the diffusive dynamics of a recombinantly expressed non-standard protein sample is studied combining X-ray crystallography, quasi-elastic neutron scattering, dynamic light scattering, and MD simulations. A key challenge here is discerning the protein internal diffusive dynamics from those of the ligand, because to ensure the saturation of the protein binding sites, a large excess of the ligand must be present in solution. Therefore, although the EISF seems to undergo a qualitative change upon ligand binding, this apparent change still has to be determined with higher accuracy. Following the addition of the ligand, an apparent elastic signal in the neutron time-of-flight data is seen as well (Fig. 3) as the first and the second Lorentzian contributions associated with internal protein motions for the liganded sample. In contrast, for the apo-ADC sample we only see one significant Lorentzian contribution from internal motions, which is very narrow. Overall, the interpretation of the impact of the ligand on the internal diffusion remains limited at this stage. At present we do not have data with deuterated d-serine as a control sample to better discern the contributions from the bound d-serine and from the protein itself to the internal dynamics. On the other hand, since most hydrogens in the d-serine are exchangeable, we effectively employ partially deuterated d-serine subsequent to solvent exchange.

By combining the QENS data with information from the protein structures, HYDROPRO results, calculations of the radial hydrogen density distribution functions based on these structures, and theoretical predictions of the short-time self-diffusion of colloidal hard spheres, an interpretation of the measured center-of-mass diffusion of the ADC protein in aqueous suspension in the presence and absence of the d-serine ligand is possible. From the IN16B data combined with the DLS data we find that these experimental diffusion coefficients follow the same trend as those predicted from calculations, but differ quantitatively. The calculations were made based on the assumption that the ADC tetramer is the protein assembly present in solution. However, the observed deviation provides evidence that larger objects than the ADC tetramers determine the diffusion. The information on the exact size of these objects is at present still limited. In the dilute limit accessed by HYDROPRO calculations, the tetramer size increases by on the order of 10% due to the ligand binding (*cf.* Table S1 in the ESI†). DLS indicates a significantly larger hydrodynamic radius of the diffusing objects compared to these calculated values for tetramers for both the apo and liganded forms of ADC (*cf.* Table S2 in the ESI†). The difference may be explained by cluster formation. QENS indicates an even larger increase of the size of the diffusing objects compared to tetramers (Fig. S13 and S14 in the ESI†), but further conclusions are limited by the absence of knowledge on the cluster shape. Further work will be needed to determine how stable these larger assemblies are, as the time-scales accessed in this study are milli-seconds (DLS) and nanoseconds (QENS), and how relevant the oligomers are to the biological function of this enzyme. Gel filtration studies show that the dominant species in solution is the ADC tetramer,^77^ suggesting the higher order species formed here are only transient, or are caused by crowding, while gel filtration involves dilution.

It seems that ligand binding affects both the nature of these oligomers or clusters, as well as the internal protein dynamics of ADC. In general, ADC appears rather stiff on the pico- to nanosecond time scale, consistent with the crystallographic data (Table 1). Binding of d-serine to ADC causes a change in the conformation of the C-terminal loop (Fig. 1), that is observed in the crystal structure, MD simulations and, indirectly *via* a change in the dynamics, in the QENS data. Other studies have suggested that the more dynamical a region is in a protein, the more influence it has on the propensity of the protein to aggregate as a result of unfavorable entropic terms.^78^ It is therefore not surprising that small structural changes in highly dynamic regions of the protein, such as the C-terminal loop in ADC, can potentially cause larger changes in the aggregation dynamics of the protein.

Regarding the fixing of the fit result from the IN5 data as the broader contribution in the fit of the IN16B data, this fixed linewidth is broader than the accessible energy range of IN16B for most *q* except for the lowest *q* values. For this reason, the IN16B fit results are quite insensitive to the exact value of this fixed width, but we refrain from definite conclusions on the internal dynamics of the apo sample for which no IN5 linewidth is available.

*R* in the EISF (eqn (8)) can be interpreted as an apparent average mean free path of the protein backbone fluctuation range (Fig. 6, top). This path is within 8 to 9 Å in the presence of the ligand in reasonable agreement with results for other proteins.^33^ Freely diffusing d-serine would not give rise to an EISF because, by definition, the EISF accounts for confined motion as opposed to the ergodic diffusion of free d-serine. Hence, the EISF in Fig. 6 can be attributed to the liganded protein. In the absence of the ligand, the IN16B signal appears too weak for a stable fit of the EISF (Fig. 6, bottom).

The MD simulations indicate an overall smaller conformational space of liganded ADC compared to apo-ADC (Fig. 2). However, with view at the weak QENS signal from apo-ADC, this difference cannot be unambiguously verified from our experimental data. Nevertheless, these data seem to suggest rather significant changes due to the addition of the ligand regarding the hydrodynamic size, aggregation behavior, and internal dynamics of ADC.

The generally weak QENS signals and the addition of d-serine, in excess to ensure saturation, pose substantial obstacles to a further interpretation. Moreover, a gap in the energy transfer ranges between IN5 and IN16B limits the connection of these data sets. Future brighter neutron sources and adapted instruments may overcome these obstacles.

## 5 Conclusions

We have reported a combined study of crystal structure and diffusive dynamics of recombinantly expressed Aspartate α-decarboxylase (ADC). We have determined the structure of ADC in the presence of the d-serined-serine ligand using X-ray diffraction. In this structure, we find that d-serine forms hydrogen bonds with some of the active site residues (ALA-75, ARG-54, THR-57) and with the pyruvoyl cofactor, and that it significantly changes the C-terminal loop. The latter finding is supported by our MD simulations. Subsequently, we have studied ADC with and without ligands in aqueous solution using both dynamic light scattering at dilute and quasi-elastic neutron spectroscopy at crowded conditions. To this effect, we have employed the newly determined structure from this work as input to HYDROPRO calculations. When comparing to these calculations, we find that the trend in the center-of-mass diffusion of the proteins in solution is consistent with the larger hydrodynamic size of liganded ADC compared to apo-ADC. However, both liganded and apo-ADC form clusters in both the dilute and crowded situations. We also simultaneously obtain information on the internal diffusive dynamics of the proteins on the scale of side-chain and backbone fluctuations. While the liganded ADC displays similar backbone diffusive fluctuations compared to other proteins with an average mean free path on the order of 8 to 9 Å^33^ at ambient conditions, no conclusive statement can be made yet regarding apo-ADC due to a weaker signal. Moreover, the spectroscopy data set is limited to just one protein concentration in aqueous solution, such that the systematic dependence on crowding cannot be studied yet. At the achieved protein concentration, the neutron spectroscopy signal is still weak, such that there is a risk of “cross-talking” between the different Lorentzian components of the model employed for the fits, and resulting misinterpretations. Nevertheless, our work points to the possibility to further investigate ligand effects in aqueous solution settings that mimic *in vivo* conditions. Predictions from the structure determination can be associated with the center-of-mass diffusion that is governed by this structure *via* the resulting hydrodynamic size and shape. The present study is limited by the scattering signal strength as well as by the available neutron beam time. Instruments at future brighter neutron sources and systematic studies including samples with higher protein concentrations may improve the information on both the cluster formation, which may depend on the concentration, *i.e.*, the macromolecular crowding, as well as on the internal diffusive dynamics. Coarse-grained simulations may help to access larger simulation length scales to explore, *e.g.*, the cluster formation.

## Data accessibility

The neutron data are permanently curated by the ILL and accessible under DOI: 10.5291/ILL-DATA.8-05-428^29^ and 10.5291/ILL-DATA.8-05-431.^30^ The coordinates and structure factors for the ADC-d-serine complex have been deposited to the protein data bank with the code 7A8Y.

## Author contributions

ARP, VTF, NH, and TS designed the research; TR, JMD, MH purified the ADC samples from *E. coli* cultures; TR, TS, JMD, SN, ARP, VTF performed the neutron scattering experiments; BAY performed the x-ray diffraction experiments; SN performed the MD simulations; TR performed the DLS experiments; all authors analyzed data and contributed to writing the manuscript.

## Conflicts of interest

There are no conflicts of interest to declare.

## Supplementary Material

CP-024-D2CP02063G-s001
