# Supplementary material for: Structure and diffusive dynamics of aspartate α-decarboxylase (ADC) liganded with d-serine in aqueous solution
Source: Phys Chem Chem Phys. 2022 Aug 11;24(34):20336–47. doi: 10.1039/d2cp02063g (PMC9429672; doi:10.1039/d2cp02063g)
Supplement: CP-024-D2CP02063G-s001 [file CP-024-D2CP02063G-s001.pdf]

Electronic Supplementary Material (ESI) for Physical Chemistry Chemical Physics.
This journal is © the Owner Societies 2022

Supplementary Information

for the article

Structure and Diffusive dynamics of Aspartate α-decarboxylase (ADC) liganded with D-Serine in aqueous solution

Tushar Raskar[1,2,3,**], Stephan Niebling[3,4], Juliette M. Devos[1,2], Briony A. Yorke[5], Michael Härtlein[1,2], Nils Huse[3], V. Trevor Forsyth[1,2,6,*], Tilo Seydel[1,*], Arwen R. Pearson[3,*]

[1] Institut Max von Laue - Paul Langevin, 71 Avenue des Martyrs, Grenoble 38000, France
[2] Partnership for Structural Biology, 71 Avenue des Martyrs, Grenoble 38000, France
[3] Institute for Nanostructure and Solid State Physics, Hamburg Centre for Ultrafast Imaging, Universität Hamburg, Luruper Chaussee 149, Hamburg, 22761, Germany
[4] European Molecular Biology Laboratory, Hamburg outstation, Notkestraße 85, 22607 Hamburg, Germany
[5] School of Chemistry and Bioscience, University of Bradford, Bradford, BD7 1DP, UK
[6] Faculty of Natural Sciences, Keele University, Staffordshire, ST5 5BG, UK
** present address: Department of Bioengineering, University of California San Francisco, USA

1 Amber99SB-ILDN force-field parameters

All parameters given below are in the Gromacs format. We followed the Gromacs manual for adding the residues:
`http://www.gromacs.org/Documentation/How-tos/Adding_a_Residue_to_a_Force_Field`

1.1 Atom types, charges and bonded interactions

The lines below were added to aminoacids.rtp in the force field folder. Atom names in the pdb need to be adjusted accordingly before processing.

```
; New residue for standalone serine
[ SSE ]
 [ atoms ]
     N    N3          -0.20564     1
    H1    H            0.25303     2
    H2    H            0.25303     3
    H3    H            0.25303     4
    CA    CT           0.03897     5
    HA    HP           0.07843     6
    CB    CT           0.05876     7
   HB1    H1           0.08320     8
   HB2    H1           0.08320     9
    OG    OH          -0.65637    10
    HG    HO           0.44926    11
     C    C            0.68350    12
   OC1    O2          -0.68620    13
   OC2    O2          -0.68620    14
 [ bonds ]
     N    H1
     N    H2
     N    H3
     N    CA
    CA    HA
    CA    CB
    CA     C
    CB   HB1
    CB   HB2
    CB    OG
    OG    HG
     C   OC1
     C   OC2
 [ impropers ]
    CA   OC1     C   OC2

; New residue for pyruvoyl
[ PYR ]
 [ atoms ]
```

```
      C     C                0.62928      1
      O     O               -0.58190      2
     C2     C                0.35434      3
     O2     O               -0.45699      4
     C3     CT              -0.12122      5
    H31     HC               0.05883      6
    H32     HC               0.05883      7
    H33     HC               0.05883      8
[ bonds ]
      C     +N
      C      O
      C     C2
     C2     O2
     C2     C3
     C3    H31
     C3    H32
     C3    H33
 [ impropers ]
     C2     +N    C      O
```

1.1.1 Addition of hydrogens

This file is necessary, if hydrogens have to be added to the pdb structure. Please add the following lines to aminoacids.hdb.

```
SLE     6
3       4       H       N       CA      CB
1       5       HA      CA      N       CB      C
2       6       HB      CB      CA      CG
1       5       HG      CG      CB      CD1     CD2
3       4       HD1     CD1     CG      CB
3       4       HD2     CD2     CG      CB
PYR     1
3       4       H3      C3      C2      O2
```

1.1.2 Translation of atom names in pdb to force-field names

Please add this to the file aminoacids.arn.

```
  SSE         O                OC2
  SSE         OXT              OC1
```

2 Additional figures on the crystal structure and MD simulations

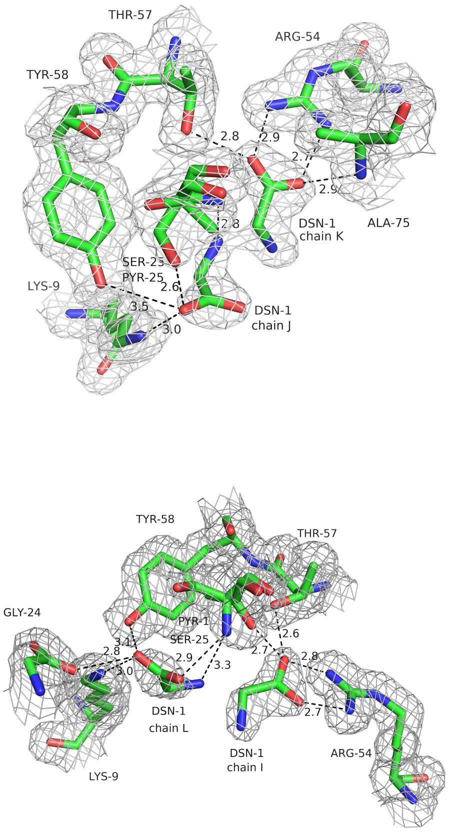

Figure S1: Hydrogen bonding network between the alternate conformations in subunit 1 (top) and subunit 3 (bottom) from the crystal structure of ADC-D-Serine complex showing the slightly different interaction patterns

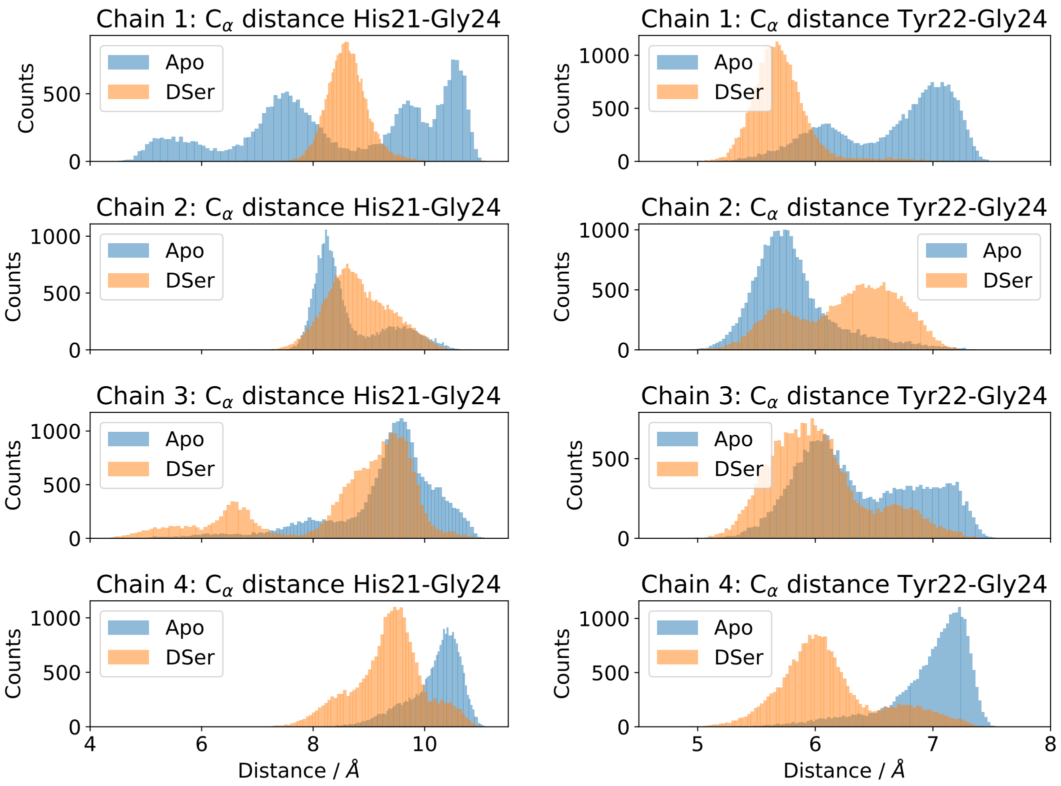

Figure S2: Histograms for the average C_α-C_α distances for the individual subunits between HIS-21 – GLY-24 (top) and between TYR-22 – GLY-24 (bottom) for apo-ADC (blue) and D-Serine liganded ADC (orange)

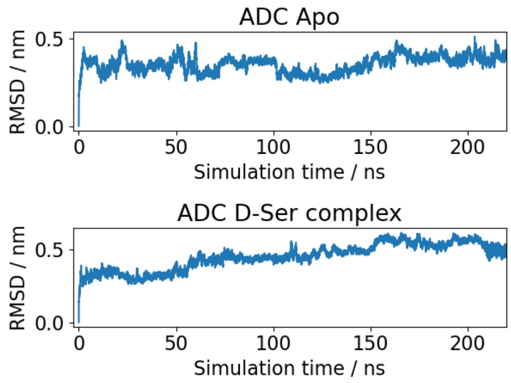

Figure S3: RMSD of the MD simulation allowing for an estimation of the duration of the equilibration.

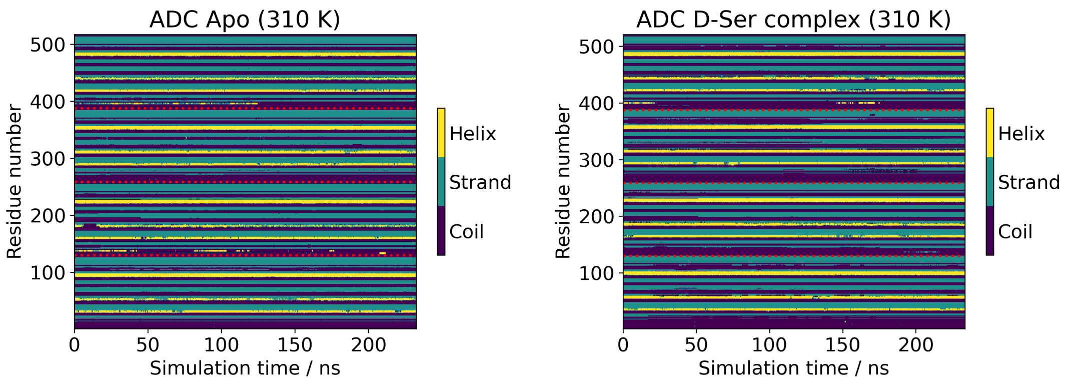

Figure S4: DSSP (hydrogen bond estimation algorithm) calculation for the trajectories of apo ADC and the ADC D-Serine complex at 310 K. The chains are separated by dashed red lines.

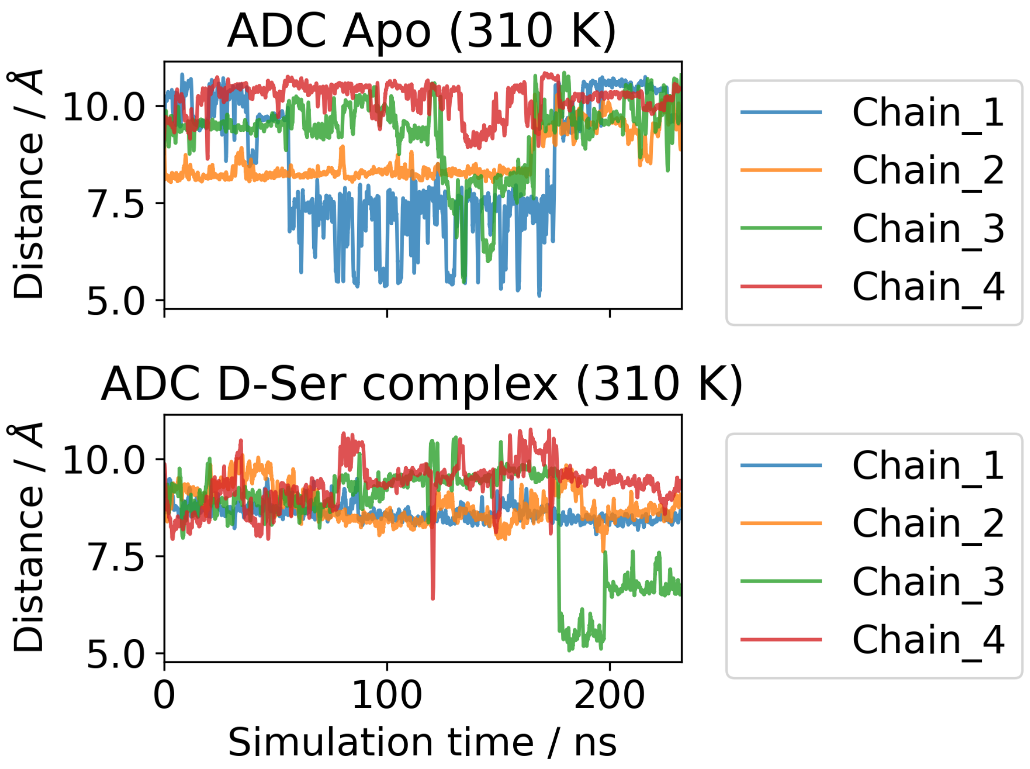

Figure S5: Distance between HIS-21 and GLY-24: the loop opens up as a result of ligand binding which involves a change in distance between Cα atoms of the terminal residues HIS-21 and GLY-24 from ≈ 7.5 to 10.5 Å (displacement by ≈ 3 Å).

3 Additional fit parameters for neutron time-of-flight spectra (IN5)

The figure S6 reports the energy resolution function of IN5 for this experiment, and the figures S7, S8, S9 report the additional fit parameters according to equation 2 of the main article. The apparent elastic contribution I_δ displayed in figure S8 is reasonably consistent with a thermal Debye-Waller (or Lamb-Mössbauer) factor of proteins that are immobile on the observation time scale of the IN5 experiment given by its energy resolution.

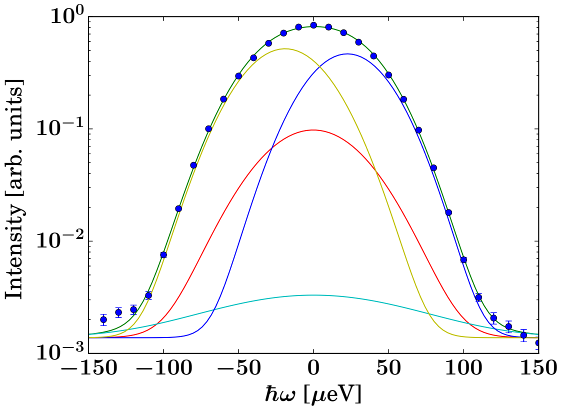

Figure S6: Energy resolution of the time-of-flight spectrometer IN5 for the employed cylindrical sample geometry with 22 mm cylinder diameter, for $q = 0.6\,\text{Å}^{-1}$, measured using Vanadium foil as the sample (symbols). The solid lines represent Gaussian functions and their sum fitted to the measured data. The sum of Gaussians is used to analytically describe the resolution function.

In the legends, ADC-lig. denotes ADC liganded, i.e. complexed, with D-Serine.

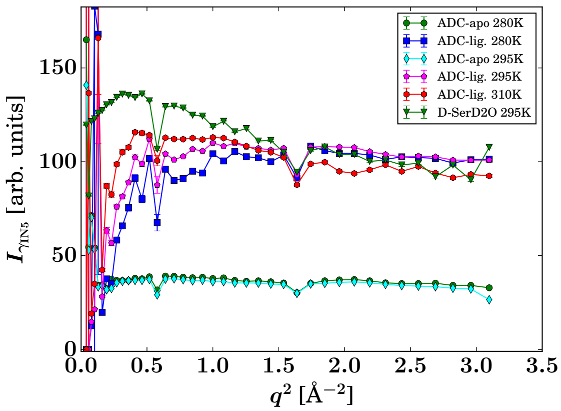

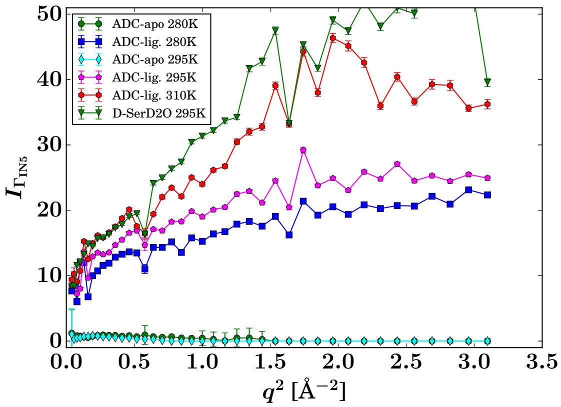

Figure S7: Summary of the fit parameters for the intensities of the Lorentzians $\mathcal{L}(\gamma)$ (top) and $\mathcal{L}(\Gamma)$ (bottom) associated with the proteins in the samples, for the samples as assigned in the legends. The error bars account for the $1 \cdot \sigma$ confidence bounds of the fits.

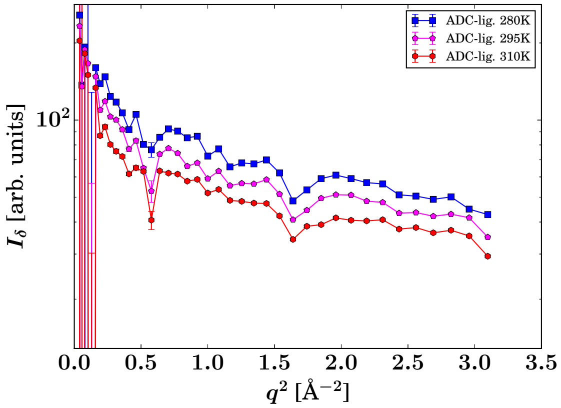

Figure S8: Fit results for the elastic contribution I_δ in equation 2 of the main article (bottom), for the samples as assigned in the legends.

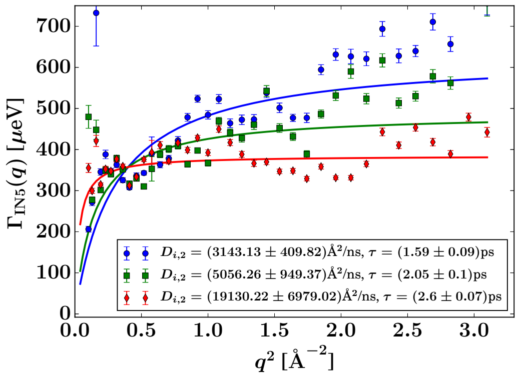

Figure S9: Width Γ_{IN5} of the Lorentzian accounting for fast internal diffusive motions observed on IN5 (equation 2 of the main part) for D-Serine liganded ADC at different temperatures (symbols) and fits using the jump diffusion model (equation 5 of the main part), the fit parameters being reported in the legend.

4 Dynamic light scattering

Using DLS, a time autocorrelation function was measured over the angular range 30-150 ° (figure S10). The correlation function for a monodisperse sample is given as

$$g^1(q,t) = a\exp(-2\Gamma t). \tag{1}$$

In case of more than one populations of clusters with different diffusion coefficients, the following equation is used as the fit function:

$$g^1(q,t) = a_1\exp(-2\Gamma_1 t) + a_2\exp(-2\Gamma_2 t) + a_3\exp(-2\Gamma_3 t) + ..., \tag{2}$$

where Γ is the decay rate and t the time. The first order autocorrelation function was treated as a monoexponential decay (equation 1) in order to extract the decay rate.

Γ can then be plotted versus q^2 which follows Fickian diffusion,

$$2\Gamma = D_t q^2, \tag{3}$$

where D_t is the translational diffusion coefficient and q is the momentum transfer defined as

$$q = \frac{4\pi n_0}{\lambda}\sin\left(\frac{\theta}{2}\right). \tag{4}$$

where θ is the scattering angle, n_0 the refractive index of the sample, and λ the wavelength of the incident beam. Due to the linear relation of Γ and q^2 (equation 3) a linear fit of the q-dependence gives the long-time translational diffusion coefficient D_t (figure S11).

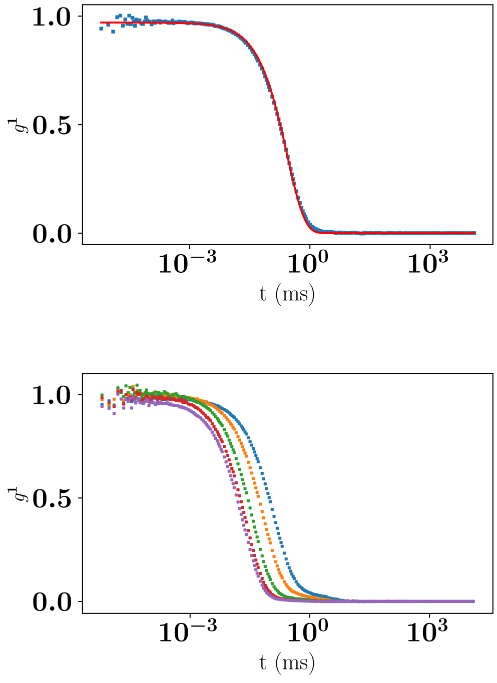

Figure S10: Top: DLS data recorded at $\Theta = 120°$ and single-exponential fit (equation 1) of the autocorrelation function g^{-1} for D-Serine liganded ADC at $c_p = 15\,\text{mg/mL}$ and $T = 298\,\text{K}$ versus correlation time t. Bottom: DLS data recorded at $70, 80, 100, 120, 140°$ for the same sample. Symbols represent the data whereas the solid line represents the fit.

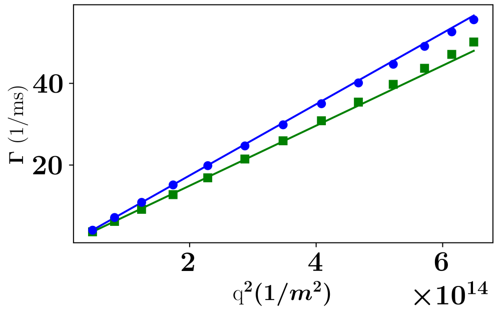

Figure S11: Linear fit of the decay rate obtained from the DLS data for D-Serine liganded ADC (squares) and apo-ADC (circles) at 15 mg/mL protein concentration

5 Radial hydrogen density distribution calculated from the pdb files

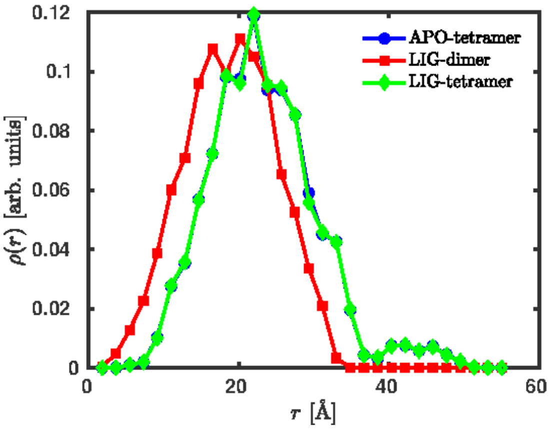

Figure S12: Radial hydrogen density distribution calculated from the respective pdb files for ADC tetramers without ligand, ADC dimers liganded with D-Serine, and ADC tetramers liganded with D-Serine, respectively, as assigned in the legend.

6 Apparent hydrodynamic radius and cluster size assuming compact spherical clusters

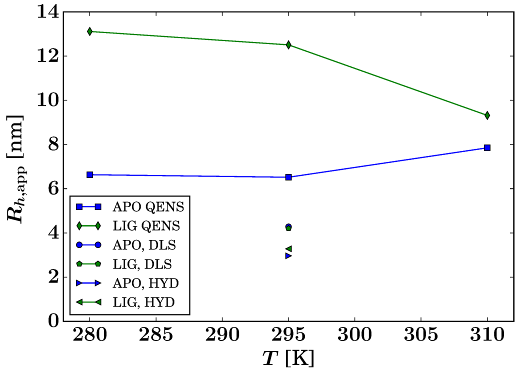

Figure S13: Apparent hydrodynamic radius $R_{h,app}$ calculated using equation 9 from the main part of the manuscript. The radius calculated from the QENS results (cf. assignment in the legend) only represents an apparent radius – which largely underestimates the actual radius –, because the rotational and translational contributions to the diffusion coefficient have not been separated (cf. figure S14). The hydrodynamic radius calculated from the HYDROPRO (denoted 'HYD' in the legend) and DLS results is exact, because these methods directly provide the translational diffusion coefficient. The different solvent viscosities in the presence and absence of excess D-Serine have been taken into account (cf. table S1 in this Supporting Information). Since the apparent radius underestimates the actual radius, a crowding-induced enhanced cluster formation can be deduced when comparing to the dilute limit (HYDROPRO and DLS).

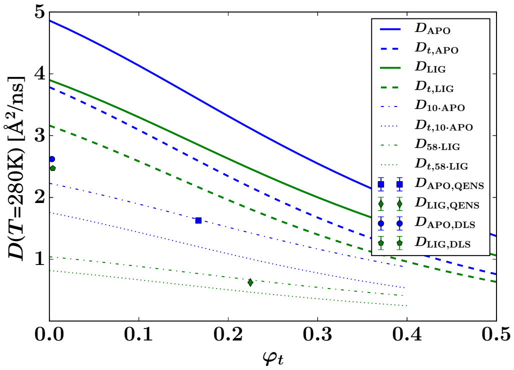

Figure S14: The considerations on the cluster hydrodynamic radius (figure S13) can be carried even further: Assuming compact spherical clusters and the radial hydrogen density distribution $\rho_H(r) = 4r_0\pi r^2\Theta(R_{h,\mathrm{cluster}} - r)$ with the normalization factor r_0 and Heaviside step function Θ (which reasonably approximates a distribution as in figure S12), the theoretical translational D_t and apparent $D = D(D_t, D_r)$ diffusion coefficients can be calculated as explained in the main part of the manuscript. To this effect, the cluster hydrodyamic radius $R_{h,\mathrm{cluster}}$ has been assumed to follow the simple volume scaling $R_{h,\mathrm{cluster}} = (n \cdot R_h^3)^{(1/3)}$, where n is the number of tetramers in the cluster and R_h the tetramer hydrodynamic radius from HYDROPRO. The above figure is identical to figure 8 in the main part of the article, but contains additional dash-dotted and dotted lines representing the cluster D and D_t, respectively, assuming $n = 10$ tetramers for the apo form of ADC and $n = 58$ tetramers for ADC liganded with D-Serine. With these crude assumptions, the experimental values for D measured with QENS can be described. The corresponding cluster radii in these assumptions amount to $R_{h,\mathrm{cluster}} = 63.8$ Å for the apo form and 127.0 Å for the liganded form.

7 Tabled HYDROPRO input and output parameters and experimental diffusion coefficients

Table S1: HYDROPRO input and output parameters for apo-ADC (pdb 1AW8) and D-Serine liganded ADC (pdb 7A8Y from this work) structures, respectively, for the temperatures $T = 280$, 295 and 310 K. The hydrodynamic radius R_h was not directly taken from the HYDROPRO output, but calculated from the HYDROPRO output for the translational diffusion coefficient D_t via the Stokes-Einstein relation (equation (9) of the main text) for $\varphi = 0$.

	ADC-APO	ADC-APO	ADC-APO	ADC-LIG	ADC-LIG	ADC-LIG
PDB file (tetramer)	1AW8	1AW8	1AW8	7A8Y	7A8Y	7A8Y
Radius of elements in primary model ($\breve{A}$)	2.90	2.90	2.90	2.90	2.90	2.9
Temperature (K)	280	295	310	280	295	310
Solvent viscosity (pure D2O) (poise)	0.01830	0.01175	0.00830	0.01976	0.01270	0.00896
Molecular weight (Da)	50391	50391	50391	50811	50811	50811
Solute partial specific volume (cmˆ3/g)	0.702	0.702	0.702	0.702	0.702	0.702
Solution density (g/cmˆ3)	1.110	1.110	1.110	1.110	1.110	1.110
Translational diffusion coefficient $D_t(\varphi{=}0)$ (cmˆ2/s)	3.784E-07	6.209E-07	9.236E-07	3.163E-07	5.188E-07	7.721E-07
Radius of gyration (cm)	2.257E-07	2.257E-07	2.257E-07	2.446E-07	2.446E-07	2.446E-07
Hydrodynamic radius R_h (cm)	2.962E-07	2.962E-07	2.962E-07	3.281E-07	3.279E-07	3.282E-07
Volume (cmˆ3)	8.235E-20	8.235E-20	8.235E-20	9.476E-20	9.476E-20	9.476E-20
Rotational diffusion coefficient ($D_r(\varphi{=}0)$) (sˆ-1)	3.174E+06	5.208E+06	7.747E+06	2.124E+06	3.484E+06	5.185E+06

Table S2: Diffusion coefficients obtained from QENS, DLS and HYDROPRO at $T = 295$ K, and hydrodynamic radius calculated from the DLS results using the Stokes-Einstein relation. The errors denote 67% confidence bounds on the fits and do not account for systematic errors arising from the choice of the model. Note that the hydrodynamic radii R_h have been calculated from D_t accounting for the different viscosities in the presence and absence of excess D-Serine in the solvent (cf. table 1 above).

	QENS $D(\varphi{=}0.09)$	DLS $D_t(\varphi \approx 0)$	HYDROPRO $D_t(\varphi{=}0)$	DLS R_h
ADC-APO	(2.82±0.03) Å^2/ns	(4.30±0.03) Å^2/ns	6.21 Å^2/ns	(4.28±0.03) nm
ADC-LIG	(1.39±0.02) Å^2/ns	(4.05±0.02) Å^2/ns	5.19 Å^2/ns	(4.20±0.02) nm

*Corresponding author e-mail

tforsyth@ill.eu
seydel@ill.eu
arwen.pearson@cfel.de
